# Effect of Percutaneous Endoscopic Lumbar Foraminoplasty of Different Facet Joint Portions on Lumbar Biomechanics: A Finite Element Analysis

**DOI:** 10.1111/os.12740

**Published:** 2020-07-08

**Authors:** Yang Yu, Qun Zhou, Yi‐zhou Xie, Xin‐ling Wang, Xiao‐hong Fan, Dang‐wei Gu, Xue Huang, Wei‐dong Wu

**Affiliations:** ^1^ Department of Orthopaedic Hospital of Chengdu University of Traditional Chinese Medicine Chengdu China; ^2^ Institution of Nurseury Chengdu University of Traditional Chinese Medicine Chengdu China; ^3^ Biomechanics Laboratory Southern Medical University Guangzhou China

**Keywords:** Biomechanics, Foraminoplasty, Lumbar percutaneous endoscopy, Three‐dimensional finite element

## Abstract

**Objective:**

To evaluate the influence of percutaneous endoscopic lumbar foraminoplasty of different facet joint portions on segmental range of motion (ROM) and intradiscal pressure (IDP) of L_3_/L_4_ and L_4_/L_5_ motion segments by establishing three dimensional finite element (FE) model.

**Method:**

Computed tomography images of a male adult volunteer of appropriate age and in good condition both mentally and physically. Obtained data was used in this study from July 2020 to December 2020, and an intact L_3–5_ three dimensional finite element model was successfully constructed using ANSYS and MIMICS software (model M1). The M1 was modified to simulate the foraminoplasty of different facet joint portions, with unilateral cylindrical excision (diameter = 0.75 cm) performed on the tip (model M2) and the base (model M3) of right L_5_ superior facet elements along with surrounding capsular ligaments, respectively. Under the same loading conditions, the ROM and IDP of L_3_/_4_ and L_4_/L_5_ segments in states of forward flexion, backward extension, left lateral bending, right lateral bending, left axial rotation and right axial rotation were all compared.

**Result:**

Compared with the intact model in backward extension, M2 increased the ROM of L_4/5_ segment by 9.4% and IDP by 11.7%, while the ROM and IDP of M3 changed only slightly. In right axial rotation, M2 and M3 increased the ROM of L_4/5_ segment by 17.9% and by 3.6%, respectively. In left axial rotation, M2 and M3 increased the ROM of L_4_/L_5_ segment by 7.14% and 3.6%, respectively. As for other states including forward flexion, left lateral bending, right lateral bending, the ROM and IDP were not significantly distinct between these two models. While focusing on L_3_/L_4_ segment, obviously changes in the ROM and IDP have not been presented and neither M2 nor M3 changed in any loading condition.

**Conclusion:**

This study provides evidence that the base‐facet foraminoplasty of L_5_ superior facet provided a higher segmental stability compared with the tip‐facet foraminoplasty in flexion and axial rotation. Meanwhile, it also shows the two types of foraminoplasty make few differences to the L_4/5_ segmental biomechanics. Besides, it does not appear to impact the stability of L_3_/L_4_ in six states of forward flexion, backward extension, left lateral bending, right lateral bending, left axial rotation and right axial rotation when superior facet of L_5_ was partially removed. These findings might be useful in understanding biomechanics of the lumbar spine after foraminoplasty performed on different portions of the facet, thus providing endoscopic surgeons a better reference for operational approach to maintain the function and mobility of the spine.

## Introduction

Symptomatic lumbar disc herniation is a common etiology for spine surgery. Although open microdiscetomy is considered to be the gold standard method, the need for minimally invasive techniques and the improvements in the use of optics and surgical instruments have led to the utilization of percutaneous endoscopic transforaminal discectomy (PETD)^1^. Since the introduction of the concept of percutaneous posterolateral nucleotomy by Hijikata^2^, Onik *et al*.^3^, and Kambin^4^, the technique of PETD has evolved over these years. PETD, by virtue of its transforaminal approach, offers several advantages over traditional open methods like shorter excision length, less blood loss, shorter hospital stay and cost, shorter recovery, lower complication rate, and lower infection rate^5^, ^6^.

As the key surgical procedure of PETD, percutaneous endoscopic foraminoplasty enables direct access to spinal canal with enlargement of foramen by resecting partial superior facet along with ablation of foraminal ligament^7^, ^8^. Since the introduction of the percutaneous discectomy by Kambin^4^, transforaminal percutaneous endoscopic lumbar discectomy (PELD) has evolved over the years and is increasingly becoming a preferred choice of treatment for the management of lumbar disc herniation. Based on the posterolateral percutaneous lumbar disc decompression, some researchers have also developed percutaneous endoscopic techniques for treatment of various kind of lumbar disc diseases, using bone trephines or an endoscopic drill and side firing Holmium:yttrium‐aluminum‐garner laser^5^. Knight *et al*.^9^ introduced laser foraminoplasty for chronic low back pain and associated sciatica, used a side‐firing laser to ablate soft tissues such as foraminal ligaments and osteophytes compressing the exiting nerve root. Schubert and Hoogland^10^ reported use of four‐grade reamers (3.5 mm, 4.5 mm, 6.5 mm, and 7.5 mm) for expanding the foraminal window by removing the ventral portion of superior facet to approach migrated discs. Although most of the practical application of percutaneous endoscopic foraminoplasty has been limited to soft disc herniation, Yong Ahn *et al*.^11^ have modified this technique and use for decompression of lumbar foraminal stenosis with a success rate of 81.8%. PETD provides sequential transforaminal passage by different size reamers, and afterwards cannula and endoscope are inserted carefully. According to relative reports on PETD^1^, ^4^, ^10^, ^12^, a 7.5‐mm reamer is the most commonly used tool in clinic to ream away the superior facet joint and the ligamenta flava for enlargement of foramen.

Although much less anatomic damage was caused by transforminal approach when compared with traditional open methods, facet joints are partially removed to enlarge the stenotic foramen. However, an unfortunate but unavoidable downside to resecting anatomical structures of the spine is an altered load‐bearing and motion environment. Many authors have reported on the biomechanical behavior of the spine after resecting partial facet using *in vitro* experimental studies. Abumi *et al*.^13^ reported that only unilateral resection of total facet made the spine unstable, while removing supraspinous/interspinous ligaments or medial facet did not affect the range of motion. Zhou *et al*.^14^ performed *in vitro* unilateral graded facetectomy on five cadavers and failed to find any significant negative effects to the lumbar stability until the range of graded facetectomy exceeded 50%. As most specimens are from elderly individuals with variations in bone quality and only motion parameters were calculated in these studies, finite element (FE) analysis, which is an alternative biomechanical model for *in vitro* models, has become a popular method for lumbar biomechanical investigations. Erbulut^15^ established FE models of graded facetectomy (total left unilateral medial facetectomy, total bilateral facetectomy, 50% unilateral medial facetectomy, and 75% unilateral medial facetectomy) to evaluate the effect on lumbar ROM. In order to get a more comprehensive biomechanical understanding of the environment in the spine after graded facetectomy, Zeng *et al*.^16^ did a further FE study which investigated the biomechanical effect of graded facetectomy on intervertebral ROM, intradiscal pressure, facet joint forces, and maximum von Mises equivalent stresses. However, these studies are based on traditional open microdiscetomy, which usually make more than half resection of facet joint, and only the effect of various resection proportion of facet joints on lumbar biomechanics was investigated. As the influence of different portions of foraminoplasty on the stability of lumbar segment is not widely explored, Yang *et al*.^17^ has conducted a clinical trial and compared two groups (156 patients) obtained respective foraminoplasty at the tip and base of superior facet. Although no postoperative instability observed in the surgical spinal unit in the 2‐year follow‐up, foraminoplasty of the tip facet showed advantages in decreasing the incidence of postoperative neural dysfunction and reducing operation time.

In order to get access to spinal canal, endoscopic surgeons use graded reamers to open and enlarge the foramen by removing partial superior facet. Nevertheless, the facet joint portion where foraminoplasty is performed depends on aspects such as surgeon's experience and disc herniation location. To our knowledge, there are few reports of studies investigating biomechanical behavior of adjacent segments after foraminoplasty performed on different facet portions. In this article, we have simulated the lumbar percutaneous endoscopic surgery and built three models (M1, M2, M3) by FE method, thus analyzing the effect of lumbar percutaneous endoscopic foraminoplasty of different facet joint portions on ROM and IDP of L_4_/L_5_ and L_3_/L_4_ level.

## Materials and Methods

### *Applicants Inclusion Criteria*

The inclusion criteria are: (i) the age of applicant is between 20 and 60 years old in good health and he has a good spirit and intelligence; and (ii) he obeys the arrangement of the research group, accepts the treatment plan designed by the research group, and signs the informed consent.

### *Applicants Exclusion Criteria*

The exclusion criteria are: participant suffered from severe spinal degeneration or severe irreversible damage of multiple spinal columns such as spinal tuberculosis and tumor.

### *Ethics Statement*

This study was conducted based on the principle of voluntary participation and was in accordance with the protocols proposed by the committee of our hospital. The patient involved in this study signed written informed consent prior to the study. The participant knew well about the study prior to the experiment and had the capability to complete all plans (Table 1).

**Table 1:** Unit attribute of lumbar L_3_–L_5_ finite element model

Component	Type	Elements	Nodes
Cortical bone	Solid187	112846	191873
Cancellous bone	Solid187	142718	286745
Cartilage endplate	Solid187	32783	65534
Annulus ground	Solid187	27584	65534
Nucleus pulposus	Solid187	30245	53317
Articular cartilage	Targel170/Contal174	1175	2637
ALL	Link10	24	32
PLL	Link10	20	30
LF	Link10	12	18
ISL	Link10	12	24
SSL	Link10	10	20
TL	Link10	14	28
CL	Link10	18	36

### *Finite Element Model of L_3_–L_5_*

**Figure 1:**
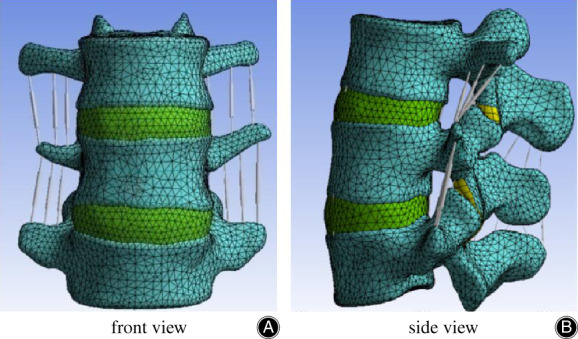
The finite element model of L_3–5_ (M1) is established by scanning the lumbar of a 30‐year‐old young male volunteer through Siemens Somatom Sensation64 multi‐sliced spiral CT (MSCT) and constructing using ANSYS and MIMICS software.

A 30‐year‐old young male volunteer was selected and examined with radiograph scanning to exclude deformity or disc degeneration in his lumbar spine. The volunteer stood at a height of 175 cm and weighed 68 kg. The scanning was conducted by Siemens Somatom Sensation 64 multi‐sliced spiral CT (MSCT), and the patient was posed in the supine position. The scanning table was adjusted to locate the scanning area, with the L_3_–L_5_ spinal segments being observed with a scanning thickness of 0.625 mm. The CT images were obtained and saved as digital imaging and communications in medicine format. The final two‐dimensional images were obtained with effect noise on the CT image, and unnecessary bone area was excluded. Then editing and removal procedures were performed on the images using Minics software. After segmentation, feature extraction, smoothing, the elements and nodes were imported to ANSYS software for remesh and obtained the finite element model of L_3_–L_5_ (M1). The model is shown in Fig. 1.

The intact model consisted of 63,8146 elements and 34,7461 nodes. In the modeling, the data for the basic geometries of the intervertebral discs were taken from average literature values^18^, and all the important spinal components, such as cortical bone, cancellous bone, posterior elements, disc annulus, disc nucleus, and endplate were also appropriately simulated. The anterior longitudinal ligament (ALL), posterior longitudinal ligament (PLL), intertransverse ligament (TL), ligamenta flava (LF), interspinal ligament (ISL), supraspinal ligament (SSL), and capsular ligament (CL) were integrated according to their anatomical positions and were represented by tension‐only spring elements with nonlinear material properties. Furthermore, the four facet joint articulations through L_3_ to L_5_ were simulated as surface‐to‐surface contact elements, a thin cartilaginous layer was created for each facet articular surface. The coefficient of friction was set at 0.1. Material properties used in the models are listed in Table 2, the material properties of the various spinal components were derived from a previous study^19^.

**Table 2:** Material properties used to represent various components in the model

Component	Young's modulus (MPa)	Passion ratio
Cortical bone	12000	0.3
Cancellous bone	100	0.3
Cartilage endplate	25	0.4
Nucleus pulposus	1	0.49
Annulus ground	4.2	0.45
ALL	7.8	0.30
PLL	10	0.30
LF	15	0.30
TL	10	0.30
CL	7.5	0.30
ISL	10	0.30
SSL	8	0.30

### *Model Validation*

The intact L_3_–L_5_ FE model was validated against the results of a previously published study by Shim *et al*.^20^. M1 was validated by the cadaveric studies previously conducted in the laboratory for a 7.5 Nm moment alone in various loading directions, with 400 N compression follower preload. After several adjustments, the ROM of the cadaver biomechanical study and M1 were compared, the ROM of M1 was always within one standard deviation of the results derived from the biomechanical cadaver measurements. Therefore, the M1 was proved to be valid and reliable (Table 3).

**Table 3:** Validation of the finite element model

Range of motion (mean ± standard deviation)				
Working condition	L_3/4_ level		L_4/5_ level	
	Shim *et al*.^4^	M1	Shim *et al*.^4^	M1
Flexion	4.3 ± 0.8	3.9	5.5 ± 0.9	4.6
Extension	3.0 ± 0.4	3.3	2.8 ± 0.4	3.2
Left lateral bending	3.5 ± 0.7	3.5	4.4 ± 1.0	3.4
Right lateral bending	3.5 ± 0.7	3.5	4.4 ± 1.0	3.4
Left axial bending	2.9 ± 0.6	2.7	3.8 ± 1.0	2.8
Right axial bending	2.9 ± 0.6	2.7	3.8 ± 1.0	2.8

### *FE models with foraminoplasty of different facet joint portions were established*

**Figure 2:**
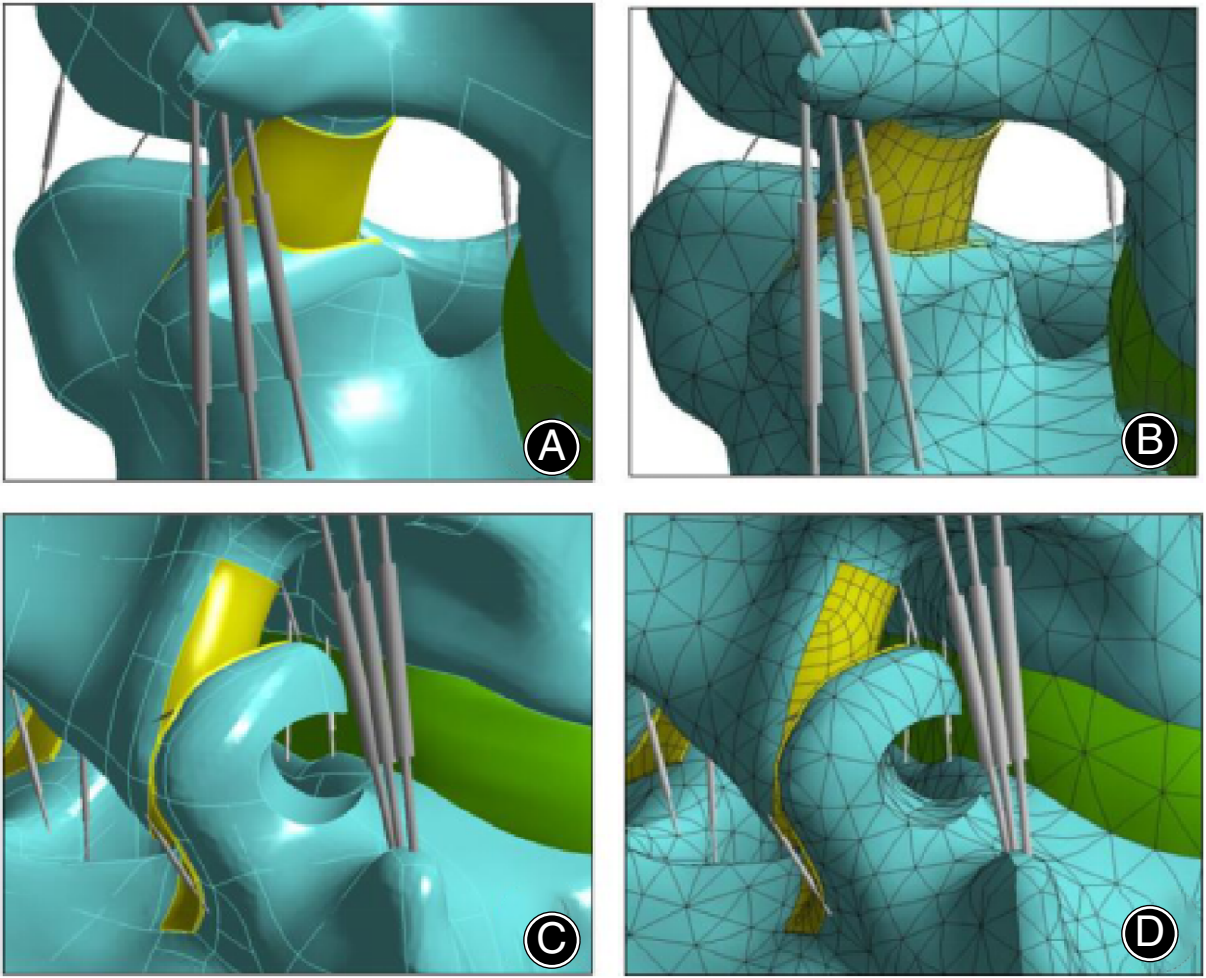
(A) Three‐dimensional FE model after tip foraminoplasty; (B) meshed FE model after tip foraminoplasty; (C) three‐dimensional FE model after basement foraminoplasty; (D) meshed FE model after basement foraminoplasty.

In order to get models with foraminoplasty performed on different facet joint portions, the intact model was modified to simulate M2 and M3 at L_4_/L_5_ level. This level was chosen due to its higher prevalence in individuals suffering from disc degenerative disease, which is the mostly performed level of transforaminal endoscopic surgery. In order to remove partial facet, surrounding CL had to be removed since they encapsulate the facets. The tip and base portion of the right L5 superior facet were marked as target points, afterwards a cylindrical excision (diameter = 0.75 cm) was made on the tip and base portions separately, which is at an angle of 30° with the coronal plane and horizontal plane, respectively. The three‐dimensional FE models and meshed FE models with foraminoplasty performed on different facet joint portions are shown in Fig. 2.

### *Load Applied and Boundary Conditions*

In this research, the inferior surface of the L_5_ vertebra remained immobilized throughout the load simulation. The L_3_ segment was physiologically loaded with 400 N. Afterward, a bending moment of 7.5 Nm was applied to the L_3_ vertebra to recreate extension, flexion, left and right lateral bending, and left and right axial rotation. All loads were chosen according to Shim *et al*.^20^.

## Results

### *Range of Motion*

**Figure 3:**
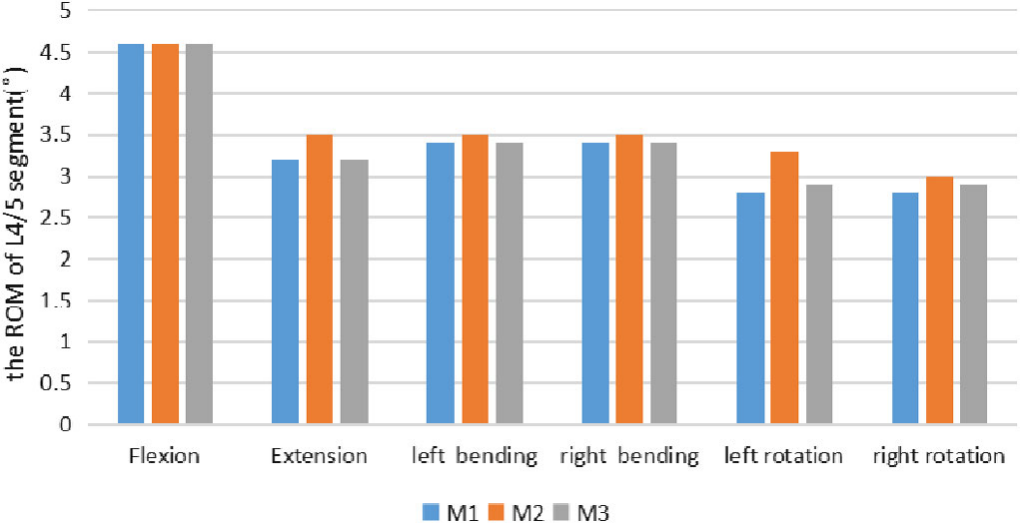
It shows the comparison of the ROM of L_4_/L_5_ segment in the M1 against M2 and M3 under a torque of 7.5 Nm in flexion, extension, left bending, right bending, left rotation and right rotation. According to results obtained by our study, for extension, M2 increased the ROM of L_4_/L_5_ segment by 9.4%, while the ROM of M3 little changed. In left axial rotation, M2 and M3 increased the ROM of L_4_/L_5_ segment by 7.14% and 3.6%, respectively. In right axial rotation, After foraminoplasty performed on the tip facet, M2 notably increased the ROM of L_4_/L_5_ segment by 17.9%, and this was the largest increase at L_4_/L_5_ motion segment among all loading cases.

The ROM of L_4/5_ was 4.6°, 3.2°, 3.4°, 3.4°, 2.8°, and 2.8° under the six conditions of flexion, extension, left and right bending, and left and right rotation in the M1 (Model 1, The intact L_3_–L_5_ FE model); the ROM of L_4/5_ was 4.6°, 3.5°, 3.5°, 3.5°, 3.3°, and 3.0° under the six conditions of M2 (Model 2, Three‐dimensional FE model after tip foraminoplasty); the ROM of L_4/5_ was 4.6°, 3.2°, 3.4°, 3.4°, 2.9°, 2.9° under the six conditions of M3 (Model 3,Three‐dimensional FE model after basement foraminoplasty) (shown in Table 4, Fig. 3).

**Table 4:** The ROM of L_4/5_ segment after different parts of formation on L_5_ facet joint (°)

Model	Condition					
	Flexion	Extension	Left bending	Right bending	Left rotation	Right rotation
M1	4.6	3.2	3.4	3.4	2.8	2.8
M2	4.6	3.5	3.5	3.5	3.3	3.0
M3	4.6	3.2	3.4	3.4	2.9	2.9

**Figure 4:**
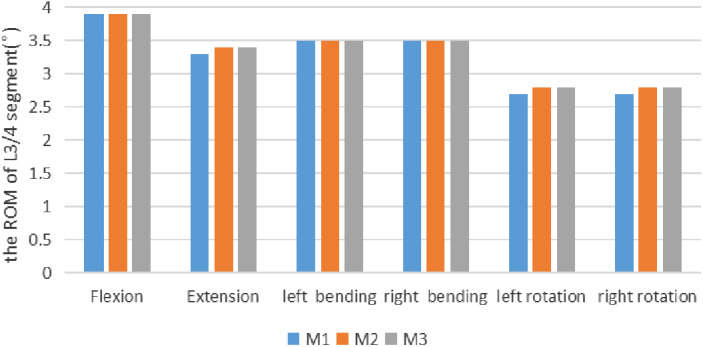
It shows the comparison of the ROM of L_3_/L_4_ segment in the M1 against M2 and M3 under a torque of 7.5 Nm in flexion, extension, left bending, right bending, left rotation and right rotation. As it is shown in figure, neither M2 nor M3 increased the the ROM of L_3_/L_4_.

The ROM of L_3/4_ were 3.9°, 3.3°, 3.5°, 3.5°, 2.7°, and 2.7°, respectively, under the six conditions of flexion, extension, left and right bending, and left and right rotation of M1 (Model 1, The intact L_3_–L_5_ FE model); the ROM of L_3/4_ were 3.9°, 3.4°, 3.5°, 3.5°, 2.8°, and 2.8°, respectively, under the six conditions of M2 (Model 2, Three‐dimensional FE model after tip foraminoplasty); the ROM of L_3/4_ under the six conditions of M3 (Model 3, Three‐dimensional FE model after basement foraminoplasty) were 3.9°, 3.4°, 3.5°, 3.5°, 2.8°, 2.8°, respectively (Table 5, Fig. 4).

**Table 5:** The ROM of L_3/4_ segment after different parts of formation on L_5_ facet joint (°)

Model	Condition					
	Flexion	Extension	Left bending	Right bending	Left rotation	Right rotation
M1	3.9	3.3	3.5	3.5	2.7	2.7
M2	3.9	3.4	3.5	3.5	2.8	2.8
M3	3.9	3.4	3.5	3.5	2.8	2.8

### *Intradiscal Pressure*

**Figure 5:**
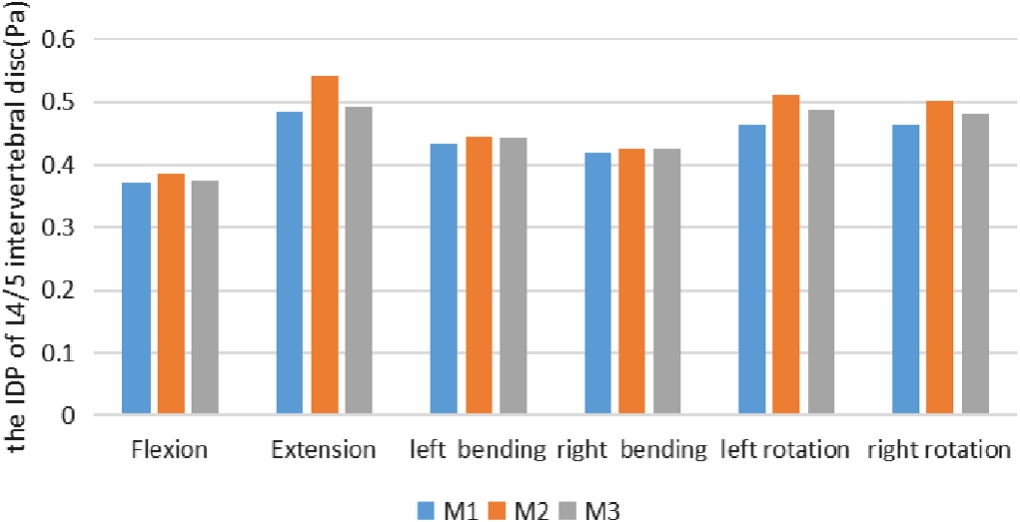
It shows the comparison of the IDP of L_4_/L_5_ segment in the M1 against M2 and M3 under a torque of 7.5 Nm in flexion, extension, left bending, right bending, left rotation and right rotation. As it depicted, in extension, M2 increased the IDP of L_4_/L_5_ level by 11.7% while M3 little increased the IDP. For left axial rotation, M2 and M3 increased the IDP by 10% and 5.2%, and M2 and M3 increased the IDP of L_4_/L_5_ level by 8.2% and 4.1% in right axial rotation.

The IDP of L_4/5_ intervertebral disc in the M1 (Model 1, The intact L_3_–L_5_ FE model) were 0.372 MPa, 0.486 MPa, 0.434 MPa, 0.421 MPa, 0.463 MPa, and 0.463 MPa, respectively, under the six conditions of flexion, extension, left and right bending and left and right rotation; the IDP of L_4/5_ intervertebral disc in the M2 (Model 2, Three‐dimensional FE model after tip foraminoplasty) were 0.387 MPa, 0.543 MPa, 0.446 MPa, 0.427 MPa, 0.510 MPa and 0.510 MPa, respectively, under the six conditions; the von Mises stress extremes of L_4/5_ intervertebral disc in the M3 (Model 3, Three‐dimensional FE model after basement foraminoplasty) under six working conditions were 0.375 MPa, 0.492 MPa, 0.442 MPa, 0.426 MPa, 0.487 MPa, and 0.482 MPa (Table 6, Fig. 5).

**Table 6:** The IDP of L_4/5_ intervertebral disc after different parts of formation on L_5_ facet joint (MPa)

Model	Condition					
	Flexion	Extension	Left bending	Right bending	Left rotation	Right rotation
M1	0.372	0.486	0.434	0.421	0.463	0.463
M2	0.387	0.543	0.446	0.427	0.510	0.501
M3	0.375	0.492	0.442	0.426	0.487	0.482

**Figure 6:**
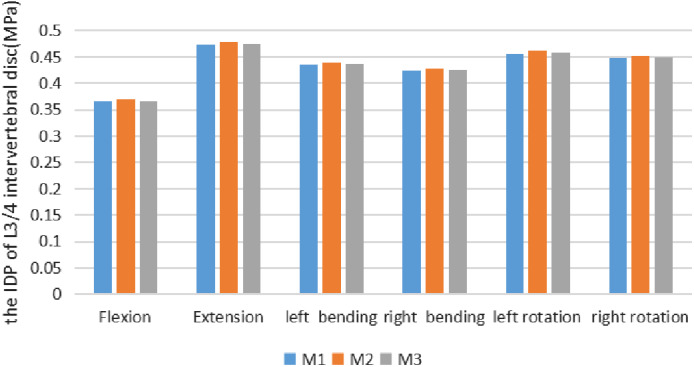
It shows comparison of the IDP of L_3_/L_4_ segment in the M1 against M2 and M3 under a torque of 7.5 Nm in flexion, extension, left bending, right bending, left rotation and right rotation. According to the Figure, neither M2 nor M3 increased the IDP of L_3_/L_4_ segment.

In the M1 (Model 1, The intact L_3_–L_5_ FE model), the IDP of L_3/4_ intervertebral disc was 0.365 MPa, 0.474 MPa, 0.435 MPa, 0.424 MPa, 0.456 MPa, and 0.447 MPa under the six conditions of flexion, extension, left and right bending, and left and right rotation, respectively; the IDP of L_3/4_ intervertebral disc under the six conditions of the M2 (Model 2, Three‐dimensional FE model after tip foraminoplasty) was 0.369 MPa, 0.479 MPa, 0.439 MPa, 0.428 MPa, 0.462 MPa, and 0.452 MPa, respectively. The IDP of L_3/4_ disc in the M3 (Model 3, Three‐dimensional FE model after basement foraminoplasty) under six working conditions are 0.366 MPa, 0.476 MPa, 0.437 MPa, 0.426 MPa, 0.459 MPa, and 0.449 MPa, respectively (Table 7 and Fig. 6).

**Table 7:** The IDP of L_3/4_ intervertebral disc after different parts of formation on L_5_ facet joint (MPa)

Model	Condition					
	Flexion	Extension	Left bending	Right bending	Left rotation	Right rotation
M1	0.365	0.474	0.435	0.424	0.456	0.447
M2	0.369	0.479	0.439	0.428	0.462	0.452
M3	0.366	0.476	0.437	0.426	0.459	0.449

## Discussion

### *Biomechanical Significance*

Compared with traditional open microdiscetomy, PETD, with its advantages of minimal anatomic damage, less facet joint removal, and operative instability, has gradually become one of the most acceptable treatments for disc herniation^20^. However, since facet joints and foraminal ligament are partially removed to enlarge the stenotic foramen by transforaminal approach, PETD still have an effect on the biomechanical property of adjacent segments to some degree^8^. For foraminoplasty, various range of proportions and portions of facet joints could be removed, depending on the surgical approaches, surgical technique, surgeons and foraminoplasty way. Although many groups^13^, ^14^, ^15^, ^16^ have investigated the effect of various resection proportion of facet joints on lumbar biomechanics, and reported that lumbar stability was not significantly affected only if the range of graded facetectomy exceeded 50%, there have been few studies comparing the biomechanical behavior of adjacent segments after foraminoplasty was performed on different facet portions.

In this research, an intact FE model (M1) of L_3_–L_5_ was constructed in this study, and ROM of the model was calculated for validation study. The intact lumbar model was validated against *in vitro* experimental studies^21^ to ensure suitability of model for further analysis. In order to investigate effect of foraminoplasty of different facet portions on segmental stability of lumbar, the M1 was modified to simulate foraminoplasty of different facet portions, by performing 0.75 cm cylindrical excision on the tip and the basement of right L_5_ superior facet elements along with surrounding capsular ligament separately. The effect of percutaneous endoscopic lumbar foraminoplasty of different facet joint portions on segmental range of motion (ROM) and intradiscal pressure (IDP) of L_3_/L_4_ and L_4_/L_5_ motion was segmentally analyzed for all six loading conditions.

This study demonstrated that the base foraminoplasty of L_5_ superior facet provided a higher segmental stability compared with the tip‐facet foraminoplasty in extension and axial rotation. According to results obtained by our study, for extension, M2 increased the ROM of L_4_/L_5_ segment by 9.4%, while little changed in the ROM of M3. In left axial rotation, M2 and M3 increased the ROM of L_4_/L_5_ segment by 7.14% and 3.6%, respectively. In right axial rotation, after foraminoplasty was performed on the tip facet, M2 notably increased the ROM of L_4_/L_5_ segment by 17.9%, and this was the largest increase at L_4_/L_5_ motion segment among all loading cases. This is similar to *in vitro* results reported by Abumi *et al*.^13^ where ROM increased significantly for axial rotation, and increase in ROM occurs in opposite direction for axial rotation. Our predicted results also show similar behavior in axial rotation. Our model predicted that base‐facet foraminoplasty also had less impact on the IDP of L_4_/L_5_ level in extension and axial rotation. In extension, M2 increased the IDP of L_4_/L_5_ level by 11.7% while M3 little increased the IDP. For left axial rotation, M2 and M3 increased the IDP by 10% and 5.2%, and M2 and M3 increased the IDP of L_4_/L_5_ level by 8.2% and 4.1% in right axial rotation. Compared with M3, the increased ROM and IDP in M2 indicated more loss of stability and a greater load through the intervertebral disc of L_4_–L_5_. This would inevitably lead to a greater risk of lumbar degeneration. Besides, neither M2 nor M3 increased the IDP or ROM of the L_3_/L_4_ segment in any loading condition.

### *Analysis of the results*

As a part of the three‐column structure of vertebrae, facet joints play a significant role in maintaining the stability of spinal motion. Facets transfer load through spinal column and restrict the motion of vertebrae, especially in the direction of extension and rotation^22^. These facet joints are typical diarthrodial joints with cartilage covering the articular surfaces, as well as ligamentous capsules that guide, couple, and limit the relative translations and rotations of adjacent vertebrae. For the tip‐facet foraminoplasty, partial bony structure was resected along with cartilage and CL, thereby violating the anatomic integrity of articulating joint, an increase of ROM and IDP is found in extension and rotation. Jun‐Song Yang *et al*.^17^ believed that, besides preserving the anatomic integrity of the lumbar spine, a nearly complete reservation of ligamental and muscular structure is beneficial for maintaining the spinal stability. Choi *et al*.^5^ has laso suggested that the resection should not involve the articular surface as preserving a larger articular surface is important for maintaining spinal stability.

Based on finite element modeling, this study demonstrated that the base‐foraminoplasty of facet provided a higher segmental stability compared with the tip‐facet foraminoplasty in extension and axial rotation. Our model predictions provide the clinician better understanding of lumbar biomechanics after percutaneous endoscopic foraminoplasty performed on different facet joint portions, and provide endoscopic surgeons a better reference for operational approach to maintain the function and mobility of the spine.

### *Limitation of the Study*

This study is more likely to be a basic study on the biomechanical effects of foraminoplasty through PETD. Therefore, it has its own limitations. In future further studies, the clinical research could embark on three‐dimensional finite element analysis of the biomechanical comparison between the preoperative and postoperative lumbar spine of patients with lumbar disc herniation undergoing PETD, which will be more valuable for clinical guidance.

## Competing Interests

The authors have declared that no competing interests exist.

## Funding

This study was financially supported by the key project grant from Sichuan Medical Association, China. No. S17024.
